# The doctor's medicine and the ambiguity of amulets: life and suffering among Bangladeshi psychiatric patients and their families in London – an interview study – 1

**DOI:** 10.1080/13648470.2013.827427

**Published:** 2013-09-02

**Authors:** Roland Littlewood, Simon Dein

**Affiliations:** Departments of Anthropology and Mental Health, University College London, Gower Street, London WC1E 6BT, UK

**Keywords:** Bangladeshis, London, suffering, amulets, mullahs, Islam

## Abstract

An interview study of 44 Bangladeshi patients and relatives in London demonstrated simultaneous trust in psychiatrists as well as in the widespread use of healing amulets. At the same time, local Islamic clerics and traditional healers were seen by many with some degree of suspicion. The authors offer an interpretation in which local healers and their methods are regarded ambivalently: the more distant biomedical framework fits with the newer modernising ‘High’ Islam (literate, scripturalist, puritanical, unitarian, urban, clerical, perhaps masculinist), as opposed to Hindu-inflected traditional Sufi Islam in Bangladesh (peasant, popular, syncretic, saintly, magical, ecstatic and possibly more sympathetic to women's experience).

When I got caught by Immigration and they sent me back, I felt they cut out my head and heart and kept it in the London hospital and I only took my body back to Bangladesh, and this is how my illness started, and that I left my heart in London.^1^

## Introduction

Cultural psychiatric research with ethnic minorities in Europe has normally promoted their alien nature: i.e. that ‘modern’ Western biomedical services use rather different categorisations of illness experience than do the ‘local’ ones of minority groups, and so that the therapies they propose and provide fail to meet the expectations and roles of those groups (Callan and Littlewood 1998). Beyond these differences lies, of course, the biomedical assumption that local understandings of illness are simply ‘folk’ and ‘personalistic’ as opposed to medical explanations that approximate to empirical truth. This is assumed to be particularly true for mental illnesses for which local interpretations worldwide do not generally provide naturalistic explanations, presumably because of the uncertain aetiology and because of the ambiguities involved in attributing agency (Littlewood 2007).

There is very little research on the understandings of mental distress and health-seeking behaviour in Bangladeshi communities in Britain. Little is known of the problems experienced by those family members who care for them, and their perceptions of access to services and service quality. Bose (1997) describes two case histories of young Bangladeshis presenting to child mental health services in east London, where their unusual behaviour was held by their families to be caused by *jiin* (spirit) possession (identified by the sudden onset of muteness, refusal to eat, shouting, swearing, disrespectful behaviour, pressured speech and visual hallucinations). She points out that it is common for Bangladeshis with such disturbed behaviour to consult traditional healers and clerics. Dein, Alexander, and Napier (2008) examine conceptualisations of ‘mental illness’ in this group: informants frequently invoke possession by *jiin* as causing mental distress, and resorting to ‘traditional healing’ is commonplace. Ahmed (2011) examines how psychotic illness is experienced by Bangladeshis in England, as Wilce (2004a) does for one woman in Sylhet. This present paper and its companions (Littlewood and Dein 2013a, b) are concerned with investigating further how one particular group – Sylhetis in Britain, both born in Bangladesh and their British-born children – experiences states characterised by biomedicine as mental illness, how they understand and deal with these states, and also the group's satisfaction or otherwise with the government health services provided (all informants had some contact with British doctors). Whilst not strictly ethnographic, this paper places particular emphasis on respondents’ own statements. The social and economic situation of London Bangladeshis is briefly summarised in Gardner (1995, 2002) and Eade and Garbin (2002).^2^

There are approximately a third of a million immigrants in Britain from Bangladesh (54% of them in London), and over 95% of them are from the north-east district of Sylhet, which abuts the Indian state of Assam, and is an area with a land holding or tenant peasantry rather than the large estates elsewhere in East Bengal (Gardner 1995). The earliest migrants to the UK were *lascars*, unskilled or semi-skilled seamen, who jumped ship and settled in London in the 1960s, acting as brokers for relatives who obtained employment vouchers for factory work. With the cessation of Commonwealth primary migration, entry to Britain has since been restricted to secondary migrants (family members). Compared with other South Asians, Bangladesh families were reunited rather later and in a sense they reflect their homeland more directly: more recent primary emigrants go to Saudi Arabia and the Arabian Gulf (where they cannot obtain permanent residence). Sylhetis are largely Sufi-influenced Sunni Muslims (10% are Hindu), most of whom follow an Islamic path which is characterised by ‘purist’ (clerical, scholarly) Islam as being contaminated with Hinduism – with reverence for (if not actual worship of) saintly *pirs*, a more thaumaturgical approach to the problems of social and religious life, and the local practices of music, dancing and oblations. Marriage and kinship patterns are traditionally through male-descended lineages, with a historical preference for arranged patrilateral parallel cousin marriage, and constant alternation between nuclear and joint (parents, sons, sons’ wives and children) households. When the father dies, his land is divided equally between the sons (with daughters receiving half a share). Until recently, women were restricted to a household role. In Britain such groups settled particularly in the East End of London where many work in ‘Indian’ restaurants and garment factories. (In the sample for the present study, anyone who cited a current job mentioned a restaurant.) Bangladeshis are considered to be the poorest and most isolated minority community in Britain (Gardner 2002, Eade and Garbin 2002). Many wealthier Bangladeshis are able to practise a more ‘purist’ form of Islam: female seclusion, religious observance, as well as religious and secular education.

The 25 identified patients (16 male, 9 female) and 19 carers (all relatives, 3 male, 16 female) were interviewed for around an hour each by one of two Sylheti-speaking Bangladeshi professional community workers, both young women who had previously completed an MSc with the principal authors in medical anthropology and health (see the Acknowledgements). One woman interviewed was both a registered patient and also a carer for her brother, and other carers were receiving counselling or other psychological support. The ages of the patients ranged from 27 to 65 years, and of carers from 18 to 73 years. A clear majority of the total sample was born in Bangladesh, 40 out of 44, but two patients and two carers were born in the UK. All, both British and Bangladesh-born, except one, had some first degree relatives living in Bangladesh. To try to tease out any local differences in interpretation for what biomedicine would term physical and psychological illness, the study included patients with physical illness (seven instances) and carers for eight patients with physical problems or somatic symptoms as well as purely ‘psychological’ illness (29 instances, including two who also had physical illness). Most informants had some schooling with an average of 6.2 years, two had none (one of whom had ‘Almost nothing – I did go to school for a while’), three had an undergraduate degree or diploma, one an MSc. In three cases, the educational level was uncertain. All respondents were Muslims and at home spoke Sylheti, the language for all but two of the interviews. About half the women respondents spoke some English. The others used their children for interpreting outside the home when dealing with hospitals, schools or the local council officials.

A convenience sample, informants were found by random at two mental health day centres and one social services disability support centre. They were interviewed confidentially in Sylheti (two in English) following a loosely structured schedule emphasising the presumed nature of the problem, its origins and causation, what help had been sought, and the consequences for the individual and their family. All interviews were recorded and then transcribed later into English. In three cases, the audio taped interview was succeeded by a lengthy conversation, which was subsequently written up. No one contacted refused participation or recording, and respondents were each paid a modest sum of £20. Quotations here are from the English transcriptions subsequently read by the authors and agreed with the two interviewers. In this paper, the hospital diagnosis has not generally been correlated with the local understandings, so the responses are just a general account of what might be called ‘distress’: this is justified by the frequency of change of medical diagnosis and the respondents’ own uncertainty about specific causative aetiology. The respondents include a mixture of people a psychiatrist would define as psychotic, as simply psychologically or physically distressed, or as quite well.

## On the doctor's diagnosis

Patients and carers are broadly in agreement with the medical diagnosis (however vague) and treatment, particularly when the patient had been ill for some time. They either couch their own understandings in terms of the official diagnosis or in subjective day-to-day understandings. Some explanations are straightforwardly ‘psychological’ and implicate worrying. Few are purely somatic. ‘I have been to so many doctors and they tell me it originates from worries and the rest is perhaps from God’, says the wife of Karim, a restaurant cook, ‘He [Karim] is very soft hearted and a simple and straightforward person. He worries too much about anything. Probably he used to worry about the growing family and how he would maintain the family. He did not tell me about his worries but I could see it.’ One patient, Najmul, goes for a more dynamic explanation: ‘When they say not to bottle up your anger because you can explode, my problem is I do bottle up my anger, so when it explodes, it explodes.’ On Bangladeshi recognition of something akin to stress see Patel (1994), and on Bengali differentiation of this from insanity see Bhattacharyya (1986) and Callan (2005, 2012).

Many informants mentioned the *daktar* (doctor) had used the English word ‘depression’ either to describe the problem itself or as a more remote contribution. Najmul continues: ‘Some time ago the doctor said my illness is called *Depression* [in English]. They have given me some medicine. Initially they gave me sleeping pills for a while. Just after taking them my body used to feel strange. I used to feel drowsy all the time. At present they give me the depression tablet to take in the morning or to take before going to bed.’

And his wife Amina adds:

There are lots of people who have this illness. Sometimes it's the wife who has it and the husband is well; sometimes the husband's got it and the wife's well. Did you know that everybody in this country has got Depression? I think everybody's got depression! In every family. Doctors come on television and talk about different diseases. I have a good understanding of depression. Depression means people talk too much, their mood is bad, they don't like anything. They tell lies not knowingly. They say the wrong things.

Some informants implicate social isolation as a cause of distress. Abdullah's wife was persuaded by her mother and brothers to leave him:

I have become totally mad because my head doesn't work. Why, I don't understand myself. For 8–9 years I am on my own: being alone is not being human and is no life. The doctors just say to come for appointments and advise me to take medication. No, they have not given it a name: they just tell me to continue to take the medicines … they say it might have been caused by Depression [in English]. As husband and wife we shared much happiness. When my family were separated I started worrying, worrying. For misfortune to come it doesn't come by notifying you with a letter or anything, it just comes. I never imagined something like this would happen to me.

About half the sample were quite vague about the precise intervening process between social events and personal distress. For example: ‘The brain does not function properly, that's all I can think of.’ But most families were confident with the doctor's eventual assessment, if only from experience of what happened after the patient neglected taking medicine: ‘she has to have the medication. As one of the doctors said, “It's like diabetes: you have to have a medicine constantly”.’

Some admitted to being fairly unknowledgeable both about the doctor's opinion in Britain and of ideas akin to insanity back in Bangladesh: one woman, who was uncertain of her age, had come to London from Bangladesh four years previously and on prompting by the interviewer said:

‘mental illness’ [*manoshir oshubidha*]? I don't understand that. If there is anything like that [i.e. one's mind not working – offered by the interviewer] back home then I don't know about it. I wouldn't know what is coming to someone else's mind, and I couldn't say what is my own mind, so how can I say? Oh, *pagol* [mad]? I hear of those things, that this or that person has gone pagol. But we don't go near them, or get to hear about them.

But one man, Fahed, in Britain since he was two, was more precise and he allied his explanation in English with that of the doctors:

I suffered from Schizophrenia [in English] which is a mental illness, which has like delusions, paranoia, seeing things, hearing voices. Paranoia is thinking something that is unusual, delusion like, thinking the same things, like something wrong with your mind, you thinking something unusual. Well, basically with mental illness, if you keep saying to the doctors ‘no, no, I'm not ill …’, you have to wake up, to accept the truth there.

And another describes experiences that would be familiar to a psychiatrist, but here with a novel (double or triple) aetiology.

I got married in Bangladesh. I was alright, thinking about how to bring her to this country. I didn't know what it was. I had learning disability and from that I started hearing voices. And this is called schizophrenia and we have medicine for it. I don't know. I still have it but no voices. Like I am walking, or sitting, suddenly schizophrenia comes. It is like a voice wants to come into the mind but doesn't come. Like from the TV, it seems like it is going to talk to me, like it wants to talk to me like. Like it is waiting there to talk to me, but one hour later it gets better. It is then like nothing has happened. When it comes, I start worrying about what is happening to me. I worry, ‘what is happening to me?’ Before then I didn't have anything like schizophrenia, I just had learning difficulty, used to worry about working. I had a diagnosis of schizophrenia, but I didn't know, until I had a fall, what it was.

## More particularly local explanations and treatment: *jiin* and the use of *tabij*

Most respondents make a loose distinction between the ‘doctor's illness’ (and treatment) ideally to be justified naturalistically by a brain scan or similar, and with the ‘mullah's illness’ (‘spiritual’). ‘Yes, one person says one thing, another person says something else. You can't believe everyone. His is not a mullah illness [i.e. not a spiritual illness], he has a problem with his brain. This is from his body, it is not to do with *ufri’.*^3^ Both doctor's and mullah's illnesses are distinguished from a mental handicap,^4^ which is seen as developmental. When problems first started or are quite unintelligible, peoples’ explanations often favoured *jiin*^5^ possession or sorcery, and hence appropriate treatment. These were often associated with but distinguished from biomedical and physiological interpretations:

There are some people who hear voices that could be jiin [that] have taken residence in them or else they suffer from depression. I am not a doctor, I can't say for sure. But nobody is ‘mental’. It is a hot thing, the blood heats up and goes to their head. That's why scientists say they are mental: they have called the blood heating up ‘schizophrenia’, and if you take medicine it cools your head down. But when you take medicine for a long time it can destroy your brain.

Extending his rather naturalistic argument and warming to the technical discussion with the community health worker, Najmul adds: ‘When [depressed women] have sex with their husband, they don't enjoy it, they don't become wet: the liquid collects and collects and then can cause diabetes.’

Whilst naturalistic explanations coincided quite happily with more distant ‘spiritual’ ones, perhaps surprisingly, patients with (what sound to a psychiatrist like) psychotic symptoms were less keen on using ultrahuman rather than biomedical remedies: ‘I don't like going to see a *mullah* [a clergyman with at least three years study at a Qur'anic school] and paying them twenty to thirty pounds. I just cry aloud to Allah.’ Occasionally, what seem to be psychotic experiences are interpreted through local understandings.

Actually, you know, I have female fairies^6^ that are kind of with me, they are always near by, and I am always chasing them! But since putting the *tabij*^10^ on, they cannot come near me. That's what the *measaab* [scholar at a Qur'anic school] has said. You know, it's like all they do, is play with me, and I am playing with them. But I can't catch them and they can't harm me, because I have God's shade upon me, he protects me, otherwise they would have killed me.

Sorcery can reach one from afar.

If somebody does *jadu*^3^ from Bangladesh, they do it via jiin or other spirits. If they send a *shaytaan*^7^ from there via a jiini, they can make you mad here. It is sent like the way you send an email. They read from a book to call the jiin to them. The jiin will come then [and] they can finish you off from afar.

A woman with a ‘depressed’ (doctor's diagnosis) husband, Jahed:

Nothing bad happened because of me. People say that somebody has done jadhu or that a ghost^8^ or other spirits have influenced him. He became ill all of a sudden you see. I did not realise it at the beginning. I realised later that he had become jealous of me. I cannot say for sure whether it was caused by worries or was from God or caused by any jadhu.

Expert advice on spiritual illness should be provided by an Islamic clergyman. One husband says:

It just happened, for some reason. Like people say it's like it could be shaytaan [possessing her] when she was at the wrong place at the wrong time. I went to the mullah to ask what's happening and he said, ‘Oh, probably your wife's got ghosts controlling her or a spirit.’ They did ask for money but only if I took my wife but she wouldn't go [at first]. They said ‘Give anything from your heart. I've got a *madrasha*^9^ in Bangladesh where a hundred people are studying. If you want to help, this is the picture, have a look.’ I think I had about £5 or £10 on me, so I said ‘That's it’.

‘Look, I have so many tabij’, says Abdul:

My brother gave me the tabij from Bangladesh. I went to the East London Mosque and got a tabij from somebody. He is always in the mosque, if you see him you believe in him, then his tabij would help: he is very fair of complexion. I gave him £20 and got the tabij. Some people in this country don't like tabij. We should have the belief that Allah will help, even if you are mad, you have some faith Allah will help. Medication helps, that's how the world is running. The Quar'anic stuff, whether it helps or not, it would not have any bad effect. Nothing bad will happen, it will be good. Even if it doesn't help, it wouldn't make it worse. Medication can either make it worse or better.

For some people the amulet is effective. ‘The tabij seemed to work a little, helped to reduce my sense of fear at night. I don't get frightened as much.’ And ‘For the mental health difficulties, the water and the oil^11^ offered by the mullahs, and the tabij are more helpful. If I see that it's a kind of illness that mullahs treat, then I will suggest to them to go and consult a mullah; if I see they have a back or stomach pain then I'd advise them to go and see their GP. The medicine from the doctors seem to offer an immediate relief, and the other things: well, they are blessed by God, so it takes time, but if you have faith in it, it will work. It works slowly…’

If you were to have a stomach ache then if you take recited water,^11^ Allah would pardon/relieve you. But I don't know what he [her husband Taj] has. What are you going to tell the mullah, what can they give him? Not everyone can understand his illness. I took him to the doctor to get his brain checked, to see if there is anything wrong with his brain. If there is no problem with his brain, why then does he act like a baby? What a two year old can understand he is unable to, that's what I would like to know. Sometimes we think it isn't to do with his brain, he [simply] has low intelligence. [But see Note 4.] You can know when someone is clever and someone is not, he has very low intelligence. My parents even used to say our son-in-law behaves like this and that. I told them he is mad, but they said he isn't mad he is just dumb, low in intelligence.

Most family members have had similar experiences.

Even the measaabs [mullahs] have said it's magic and when I go out, it's like I see them [jiin] … When I go out, there are girls following me and calling me, but when I look back, I can't see them! So you see, it was black magic. And when I went to the mosque, I was given a tabij, and was given water that was blown over, and after that, those things can no longer come.

And another relative comments:

Bengali [malevolent] magic is *bhut lagi-gese* [a spirit that has possessed you or is after you]. But a *phunga-jiina mumin* has got to me now. When I went to Bangladesh I probably urinated or something on their path, whilst they were travelling, so they got really angry with me, and so to punish me, they are now harassing me, making me restless. I have asked them to forgive me, and I said you know, even if I have done it by mistake, please forgive me, and leave me. But they said that they will return after two years. But I do not know why they will come back in two years.

Well, if Shaytaan^7^ is going to do something [to cause the symptoms], or whether it is something due to me [from God for his own actions], there are cures for both of these. For example, if you went to bed, and had breathing difficulties as a result of Shaytaan having attacked you and holding you down, then, you can recite prayers from the Qur'an, and then it will leave you – you will have healing. Well, you will see images of something trying to kill you; you will sense it; you will see awful looking creatures, and you will be frightened that they are trying to kill you. So, if you want to successfully liberate yourself from that, you will have to be *pak.*^12^ You will have to go to bed in a pure state, then of course none of these entities can come near you; that's according to our religious perspectives and beliefs, but it's not something adhered to or believed by people in this country. How can they, they are not Muslims, they do not believe in the Qur'an.

He continues:

I recite prayers from the Qur'an and other Islamic books that I have learnt, and I ask God to help me, and sometimes I do receive help from God, when I am frightened I do receive help. It used to happen to me about six months ago, I used to feel quite scared, like someone is trying to capture me, kill me, but I came out of it, God helped me. For example, at night I feel that someone is trying to suffocate me to death. Then I will purify myself with a ritual [called *wudu*], and will recite Ayatul Qursi [the Verses of the Throne from the Qur'an] seven times and blow over my chest, and then I see that nothing tries to suffocate me during that night. It is stated in the Qur'an, that efficacy of these prayers depends on one's state of soul [glossed by our interviewer as sincerity, level of trust and belief in God, and adherence to religion].

Only one woman, Amina, claimed some healing skills of her own.

I too undertake some rituals undertaken by mullahs, I have some of that knowledge. There are particular difficulties that are faced by women, and if they undertake certain rituals they find relief and healing. For example, last week a lady came and requested me to give her some blessed water, I asked her why and she said to me that she wanted to have a son. I then taught her some prayers: verses from the Qur'an; and I instructed her as to how she should purify herself, and how she needs to go to bed in a purified state and sleep on her right side, and she should present her request to God.

Whilst not decrying spiritual explanations, in many cases some people are doubtful of an ultrahuman explanation in a particular case depending on the efficacy of spiritual treatment.

My father-in-law got him [the husband] checked out by so many doctors in Bangladesh, then took him to a *pir*^13^ and after that he got better. He used to throw the furniture about and beat people up. He was severely unwell. The pir got him to sit under a tree and walked around him seven times then recited something and blew over him. Under the tree there is a pond, he got him to dip in the pond seven times and gave blessed water. The pir also instructed that someone should at three o'clock at night get a container of water from a large river-like pond. But no one is brave enough to go at this time of night. At this time there are so many spirits around the pond. Not many people would brave this. The person who should go needs to be pure and pray their prayers. This type of person needs to go to fetch the water. His uncle got this water from a pond. The pir recited over this water and got him to dip in the pond under the tree, seven times. And then broke a slim twig from the tree and beat him from the head to feet and then head to feet. The pir said something has possessed him. You know they say these kind of things in Bangladesh. Whether it is true or not, only God knows, I don't know. After this, when he came home, he became better. My father-in-law brought him to the UK, and then [again] to Bangladesh, and he would take him to the pir if his symptoms returned. He would get better after seeing the pir but then again would get ill. The pir said if he was possessed this would go away. But this illness is from his brain as he is getting unwell again now. I think he was born with it. The pir thought this [was possession] since he was beating up people indiscriminately, in Bangladesh people usually do that when they are possessed by jiin and going to the pir makes people get better. [And] this doesn't come back if you are possessed. His [illness] is lifelong, it has mixed with his blood. When he gets medication and injection this gets better. I learn so much about mental illness from him. If he doesn't have the injection for two weeks he will pace up and down and doesn't sleep. And if he gets the injection he sleeps well.

In this case, the limited treatment effects result in a reconsideration of the pattern of symptoms.

## Spirits in England and Bangladesh

A patient with physical disabilities at the London day centre describes his own granddaughter's recent upset:

Ufri is when you come across the air touched by *bhut peroth* [evil spirits], things like that. For example, last Thursday my 13 year old grand-daughter woke up screaming and crying and she wouldn't stop: we all woke up. Then we called upon a mullah that we knew; we didn't call the ambulance. The mullah came and then he gave some oil and water which was blown over by him after some recitals [of Quranic verses], and he gave a tabij too, and her distress stopped. She wasn't able to breath, so we asked the mullah why this was happening, and he said that this has been caused by air touched by the spirits. It was only three days ago that we had visited the park, and we thought perhaps that's where this has happened; we haven't yet understood where this had come from. She is still at home, not attending school, as she is so scared, she won't even go to the bathroom, she is so scared! The mullah gave *pora pani*^11^ and pora oil [oil blown over with recitals], he also blew over her [gave her a *foo*], and also a tabij. The mullah is also a relative of ours, he is her maternal cousin. He came and stayed 3–4 days; when he stays she appears to be less frightened in the house. Lately she is sleeping with her mother, she used to sleep with her older sister before but she doesn't anymore and she is frightened of being alone anywhere. Now, how are you supposed to let her go to school, when she is so scared, how is she supposed to walk alone to the school? The following day, we called the ambulance and the staff asked her many questions – she can speak English; they took her to the hospital, undertook many tests, but they could not find anything! Well, she was screaming at 2am in the morning! She was screaming, and she would not stop! Her mother held her to try and calm her, but she would not calm down, all she did was scream, and was crying and screaming! We all woke up! She said that she can't breathe, that she just can't breath; she can't sleep because she is scared because she can't breathe, her breath doesn't come back. Now, the ambulance did take her, but they didn't find anything either: did she have an asthma attack? An allergy attack? No – she didn't have any of these! They couldn't find anything wrong with her – they even took her to the hospital. If you even talk about this in front of her, she gets scared then too. If you ask her anything or talk anything about her, she becomes scared, so no one in the house now talks to her.

Bangladesh is seen as spiritually more dangerous than England and problems are often attributed to one's presence there:

I used to have these waking visions, of my death, you know, used to see my dead body in a coffin, and people taking me to the grave, and leaving me. That's what I used to see, when I was in Bangladesh last December. I don't know what I did, but people say that I went inside the bush areas, and where there was water. When people go from England, they are cautious of where they are walking, they do not go inside jungle areas or walk in mud, they take care as to where they will walk etc. But I used to run into the fields and here and there. I used to be on the run all day. I would pray the dawn prayers, and then I would leave the house running about. And I couldn't sleep. Even after my return here [to London], I still couldn't sleep, and even the doctors were scared, they all thought I was dead. They said they would give me medicine at home, and asked who would care for me. I said my wife. So, they come and they give me medicine, stay for about fifteen minutes, then they just go. Now my [financial] benefits and stuff is all in a mess, I don't know much English, so I just manage with what I know. Now I want someone to help me and my family.

The same man, who had what to a doctor would sound like psychotic experiences, describes how:

One day around 7pm, I went to the Mile End Park [in east London], and I saw two beautiful women sitting there. They lit a gas lighter, and drew my attention to go to them, and so I did. As soon as I went to them, they asked if I would offer them a cigarette, and they asked if I would marry them. I replied that I was married. They said that didn't matter. I said, ‘Well I don't know where you are from’. As soon as I said that, they asked, ‘what religion are you from?’ I said Islam. They asked whether it was not possible to change my religion, and I said that I couldn't. Then my whole body was trembling, and then I realised, within two minutes that they just vanished! So, how can I not believe that this is not black magic? I do believe that it is. I told the doctors all this and they said to continue with the medicine. I am also taking treatment from mullah, and the rest lies with God.^14^

There was a woman in Bangladesh who gave me a special ring to wear; I threw that ring away into the sea, the river … you know, there is one river near my road [the Thames]. I used to be able to see things through that ring; and then my whole body would shiver. And I noticed that my son could not sleep in the house. And I also noticed that when I am asleep, if I try to touch my wife's body, it used to feel as if her body was a ball of fire. That's what happened to me, due to the ring. When ever I touched my wife, her body felt like a ball of fire. You know, we are husband and wife, so we sleep together, and when I sleep next to her, and try to touch her, it's like her body is fire! Whenever I tried, it used to feel like that, so I used to withdraw.

When I went to my uncle's house, they were saying you know, ‘why are you acting like this, why are you mad, nephew, you don't even eat or anything, why?’ I said ‘Mama [maternal uncle], you keep calling me mad, but look at the sun’, I pointed out with my hand to look. We saw a person there in the rays of the sun, he looked like a *sahabi* [someone saintly, the word is used to refer to the Prophet Muhammed's Companions]. A man dressed all in white, holding a walking stick, and he was talking in the air! Whether it was Shaytaan, or a ghost, or God who showed me that vision, only God knows; no one else can tell the truth. And I showed four people who saw this with me; if anyone wants proof, they can go to Bangladesh and ask those relatives, who will confirm this. Then I said to them ‘that's it! He's gone now! You will never see him again!’ So I told them to go home, and they went, and the person had also disappeared in the sky.

I think, it was Prophet Muhammed, or may be a saint! Someone like that, maybe I am walking on the wrong path [not acting in accordance with religious teaching], and so I was shown [this] as a reminder to come back on the straight path. Maybe it's a message: you have a future to live, you have children to bring up properly, your children might lose their way on the path, if you don't try and return on the right path. I saw a lady when I was asleep. I had kept a beard because I became mad! And during that time, I remember, a lady talking to me, saying to me ‘your face looks so beautiful, it's as if you have light upon it.’ I woke my wife up, and told her. She told me to go back to sleep. During that time, my wife didn't like talking to me, if I used to say anything, she would just say other things to me instead. So when I threw that ring away, I have been feeling a lot more peace within me.

Often what seem biomedically speaking to be psychotic experiences are normalised by family and clergy – or so some who are affected affirm.

That ring was very powerful. It had power. Once whilst walking, I saw a sight, and it was telling me that two women will be talking to me. And then you know, two traffic wardens who were ladies, had come up to me, and spoke with me. They asked why I was looking up in the sky; I used to do that a lot since I saw that saint in the sky, I used to hold out my hands in prayer towards the sky; after I went to the park, and had sat there, I used to pray there, and feed the birds, I used to take bread for them. I saw so many things in Bangladesh, my wife brought me back, when I went. But the measaab said, that if I had stayed in Bangladesh for another three months, I would have been gifted with something, I would have found something extraordinary!

So I prayed. I request God to heal me, to grant me a long life, to enable me to earn money, to keep me well, to forgive me for my mistakes, and to take me upon the straight path [towards God] – that's what I used to pray. It's because of that ring I had. The measaab said that it wasn't given by a woman, it was a fairy. It's not a woman, it was a spirit that took a human form, and came to me, and gave me that ring. The measaab said that I could have asked for anything with that ring, it was so powerful… I couldn't sleep when I was in Bangladesh, I was scared for my life; I used to feel that it was attacking me, I used to feel that it's trying to kill me, it's holding me at my waist, and trying to strangle me, it tied my body, my arms and legs. So I threw away the ring, and I sought forgiveness from the spirits, ghosts – that I wanted to live with my children.

I saw a dead pigeon, and when I gave it some rice, it became alive! And when I saw another one at the Shah Jalal tomb [in Bangladesh], I saw a dead bird, I threw some rice at it, and it disappeared – that's what people say that they have witnessed me to do. And also, I saw two people dead on the road, covered in sheets, and I scattered some rose water on them, and they became alive! At that point, my cousin took me from there, and took me home. And my cousin said, you should have asked those two men for something, and you would have been given it.

Explanations of spirits are held in common by the patients and their families. If the above account seems evidently psychotic, it appears that certain aspects of it were shared with his relatives. Two of them say about themselves:

And after I came back to England, my mother had given me blessed water, oil and tabij to wear, so I feel that due to all these treatments the symptoms [jiin visitations] alleviated, and the measaab said that they will return in two years, and when they come, they will either kill me, or give me something. They said that I should offer service to people, and then people will respect me.

And:

When I was 12 years old I gave a pir my name on a piece of paper, and he saw in the [divination] water that I was going to go abroad, how did he know? And two years later, I did fly, how did he know that I was going to come to London? He said that I was going to gain *shath rajar dhon* [the wealth of seven kings], and I received four boys, so I did receive the wealth. How did he know that I was going to receive this? He said that I was going to have seven boys, well I have four, who's to say that I won't have three more? So this is becoming true isn't it? If these people were not godly and saintly, how could they say things about the future?

Some of our informants later attribute such experiences simply to a dream-like state of mind:

I used to see that someone is coming and has grabbed me, that's what I used to see. They don't look like human beings, they have really long nails, you see them in the dream state, and your mind sees these things – one is going, one is trying to kill etc. For example, if you came to my house, and I gave you a cup of tea, you might wonder, ‘has this gentleman put anything in my tea?’ [to poison or alter mind or behaviour or cause ill health]; and you will keep thinking about that, because you brought that thought in your mind; but, I haven't given anything in your tea; but it's still in your mind and you have become anxious and are feeling restless due to your thoughts, which are creating suspicion in your mind.

## Problems with relatives, mullahs and the tabij

Problems of illness are cut through with family difficulties which may sometimes proceed to sorcery. One man blamed his parents and paternal cousin:

They forced me to get married. They made me drink water which had been recited upon; this made me agree to the marriage. I didn't want to get married. I said I don't earn enough to take care of my wife and children … It happened that one day when I returned from work, I received a letter. I see it is a tabij. After touching the tabij I felt a burning sensation. It was my cousin, he wanted me to get his son to this country.

Some people found the tabij did not work. Minera comments about her husband who is ill:

After seeing the doctor the first time we had to take him again when it became worse. Afterwards we took him to Bangladesh and showed him to various mullahs. People in Bangladesh told us to take him to the mullahs. The mullahs gave him tabij to wear around the neck and arms and blessed water to drink, as well as blessed oil to use on his body. He used it as long as we were in Bangladesh for three months. The mullahs said to use it for 21–24 days, he did but we saw no benefit from it. He took the medicine along with that too. I can't see that he is improving: he is getting worse.

Others think the efficacy of the tabij is largely psychological: ‘And since I put the tabij on I am more chatty. And everybody is like it's because of the tabij you are loosening up. I don't believe that: obviously jiin and things and black magic exist, but for somebody to do ufri stuff I don't believe that.’

We took [my son] to see two or three mullahs. They all said it was koni-jadu. We also took him to the Shah Jalal and Shah Poran [Sufi shrines] but nothing helped. The mullahs don't say who did the koni. They said it would cause a fight, that's why they don't say. All the mullahs said the same thing, even though they all live in separate areas. They only said that someone did it for a good reason but it went wrong.

I don't need it, there is one God. If you do it yourself, there is no need for tabij. Using tabij is not right. Before, there were the righteous mullahs who said give money to the mosques. The ones who do tabij now, they want money for it. Yes, that's how it seems, sister. I don't believe in these other forms of treatments. It's not good to go near these kinds of treatments. I don't think it is a good idea. They are bad people [who offer tabij], they make things worse for people.

One mullah says it is koni, another says it is jadhu, another says shaytaan. Nothing helped, he [the husband] was the same. Mullah and things are really bad, that's why I don't go. The last time he went to Bangladesh, this mullah came and said to him, ‘you have koni, give me money, give me money’. He had a problem in the head then, he gave a bundle of money to him, the man asked for more money, saying give all the money you have. My husband said ‘it came to my head at that point’, but I couldn't understand fully. He said they [the mullahs] are all mad, all they want is money from the people.

The tabij itself may not merely be ineffective but it also has the power of harming depending on the person who offered/sold it. Even to consult a mullah or healer about an illness may be suspect: ‘Say I was to go to a mullah or kobiraj for him, people would say look your wife is doing black magic on you. You don't know Bengali people,^16^ they want to break up your family. They might say one thing to the husband and another thing to the wife, to cause problem in the family.’ And similarly, even visiting relatives in hospital may be dubious: ‘It is his dad and he didn't go to see him. You know how Bengali people are, they would have come and accused us.’

A wife and carer comments:

I think it is ufri or something, maybe it is black magic or some sort of possession. You know people get caught in remote areas or are possessed in remote areas. He had a take-away [restaurant] in a remote area [of East London]. He would deliver take-away during night and day. Someone from Bangladesh did it to get more from him … If this comes from Allah then I will be patient. If it comes from something else, then Allah will judge this.

‘[My husband's] brother took him to see a mullah for tabij,’ says the wife of another patient at the clinic,

The mullah said that he had black magic done on him. Someone wanted him to marry their daughter and it went the other way, something like that. I really don't have faith in this to be honest. If it is an illness, it is an illness. If people had this ability then no one could stay well. Everybody thinks the illness started after the accident, but I believe he had a problem in the brain. At the same time his father died. He was his father's favourite, that's why I feel he couldn't tolerate it. His brain was inadequate.

A few think using an amulet is too much like ‘magic’. Rushida, a British-born university graduate, objected to amulets altogether:

Basically, if you are a true Muslim and you really believe that Allah can help you, then you make *du'a* [personal prayer] for Allah to help you. If you have something on you, even if it's the words of Allah, and if you put your trust in that thing … I can't. You know I need to learn from the Qur'an, I need to teach myself things, you know like how to be a good person, morals, and how to be a good Muslim and a good person, and how to treat others well. Not to lie and stuff like that – but if I want help it would be by asking Allah, not by putting my trust in things – because that's like idol worship. If somebody wears a ‘tabij’ I don't think they have faith in God. Some people might get confused. If they put their faith in that thing then that's completely contradicting everything that they're meant to believe in.

This follows the practice of modern ‘purist’ Islam which cites the Prophet speaking against the practices of the pagan Arabs who disregarded evident natural causes in preference for mysteries and magical powers, such as charms, talismans, incomprehensible utterances and the tricks practised by sorcerers and quacks. In one *hadith* he is quoted as saying: ‘Whoever wears a charm is guilty of associating partners with God’. In another he says: ‘Whoever wears a charm, may God never fulfil his purpose, and whoever wears a talisman, may God never let him enjoy peace of mind’.

A patient who had paid a visit back to Bangladesh confesses that ‘The mullah gave a tabij that I tied around my head. But I felt my world was upside down when I wore it. I quickly took it off and cast it away into the river when we came from seeing the mullah.’ ‘If someone's heart doesn't want religious help, it's no use giving them grief,’ comments another, ‘using tabij and things is not useful. I read the Qur'an before I go to sleep and do my prayers. There is nothing else really.’

Sheftali, a depressed woman, likens their use to sorcery: ‘I only spoke to the doctor about this, I don't believe in going to mullahs. I never used tabij or blessed water. When children have ufri then use blessed oil and blessed water, but don't believe in doing jadu. I don't think there is any ufri in this country [England].’ But one man in London, diagnosed as schizophrenic, said: ‘I went to a mufti. Another mullah came to my house. I told him that I can't work or anything. He blew on me and said that I have problems in my head. I took him to my house and fed him and gave him money.’

Even when a *tabij* is requested, people complain about the mullah's greed.

Well, the GP says that my headaches are called ‘dizziness’, but of course they will say what they know. They gave me these really strong pain killers and they give me injections too. My husband got me a tabij and some blown water [*pora pani*]. The mullahs say someone has done korni or jadu. Each gives a different diagnosis; like one said I had walked in the shade of the bhut. You have to pay, for some £200, for some £300, for one tabij. It's not something negotiable, you have to pay for that. I don't know if they work. Because of my sufferings, I keep them upon me, use the blown oil, and the water. I keep on wondering ‘why is this happening to me, why is this happening to me? And then I think what the mullahs have said, and I wonder whether there is some truth in what they say, that perhaps someone has done black magic to me because I wasn't unwell like this before. They always say someone among the relatives has done it. They never say who.

One cynic argues that, ‘Mullahs don't say nothing: you go to them and tell them about your problem and they give you some blessed water from reciting the Qur'an. Without money, mullahs won't do anything.’

And Abdel mentions that in London: ‘A couple of years ago the mullah wrote a tabij for £10. It didn't work, I didn't believe in that tabij stuff. I took it off after a couple of weeks. The tabij is not going to cure me. I didn't believe in it. I tried it, nothing happened, I took it off. I didn't like it.’ In Sylhet, ‘We went to big mullahs, one took money for a cow and in return gave a tabij, but this didn't work. I went to another mullah in London, he charged £150 for a tabij, we got it but it didn't work. The mullah just gave a tabij. All the mullahs said it was ufri, or it could be mental illness. They said take the tabij and see if it might work, but they gave me no guarantee.’

His parents got the tabij and things and visited different pirs’ places, used blessed water and oil, things like that. They did this before and after our marriage. Before giving the tabij the guy sits in a trance. Only God know and he knows, I don't know what he does. Whether he is conscious or unconscious I don't know, only God knows. After this he gives the tabij. Then he tells you to form the intention of sacrificing a cow or something for Allah, so you do. He does his healing. You then see some good and then some bad. For these few years no one is bothered to help. It doesn't improve. We have given so much, given money, a cow. Whenever he went to Bangladesh, they did this. It has now been 10–12 years, he hasn't been to Bangladesh, so nothing has been done. Now he is dependent on medicine, but the medicine doesn't seem to be doing the trick.

‘I would say “Get help from the doctor”,’ comments Najma. ‘But people do not listen: they seek help from the mullah, the herbalist. They continue with this for a while, then in the long run their condition is worse. I told them to go to the doctors. Doctors don't listen immediately if you tell them once or twice, you have to go repeatedly and tell the truth not lies. They went to see a mullah but it didn't help.’

Shazna is ambivalent about the amulets but still uses them: ‘People give so many things, like blessed dates, blessed oil or blessed water. I do not get them in this country. I normally get them from Bangladesh. One of my uncles is a mullah. I do not get them here: here I do not have faith in anyone.’ Unusually, she feels safer spiritually in Sylhet, perhaps because of the family mullah.

## The doctor's treatment

Previous literature has suggested a dissatisfaction of South Asian patients with biomedical psychiatric treatments (Anand and Cochrane 2005), but our current informants are, to our surprise, generally enthusiastic about medical treatment and perceive medical staff as caring and trustworthy, in contrast to the expectation in Bangladesh itself (Callan 2012, 162). One carer has advice for others: ‘This type of patient should stay cheerful. If they are not getting better I will tell them to go and see a doctor and tell the doctor. The doctor would advise them then what they should or should not do.’

Most, patients and relatives, support biomedical treatment: ‘The GP sent him to hospital. My husband gets appointments to see the psychiatrist every six months. We are happy with them: if it gets worse they increase the dose of the medicine and tell us what to do. I have always been happy with them. I tell them I am more or less OK. The rest is up to Allah.’ One lady told her doctor about being given a tabij: ‘I did tell my GP about these treatments and then they sent me to see a psychiatrist! He gave me these tablets for depression. “Mental health difficulties”? Isn't that just the same as jadu [sorcery]? When I take the medicine I feel a little better, just a small amount. I can sit somewhere and talk to someone.’

However,

The medicine that they give, they used to have a lot of side effects that I couldn't cope with, so I told the doctor that I couldn't take it, and they agreed for me to stop them.

The doctors say it's arthritis, muscle pain, they can't do anything. I take around eight paracetamol tablets daily for the pain. I also take *sorotha* [a bitter herb] from Bangladesh and I take *neem* [herbal pills] for the pain. This pain is giving me a great deal of suffering, everything else does too but this pain gives the most. What do I do? I sit quietly and call upon God. Sometimes I think of myself that I should go and see somebody for a tabij or something but then I wonder, well – what would be the point? My illnesses are nothing to do with ufri.

Another rationalist:

I know people from our culture believe in magic; I don't think tabij can affect someone in the brain. I have never believed in that. At first [the husband's] parents took him to many, many places to measaabs and pirs. They went to many places and spent much money. But nothing helped at all. They took him to Bangladesh and went to the pir's place and mullah's place, they run around from place to place. I don't think it is a tabij, it is an illness. I have read about it, that if someone takes something in deeply and thinks about it deeply and thinks about it all the time, it affects them, their sleep and eating. This is what I understand. I read any leaflets where ever I find them, in different centres, GP's surgery. When they went to see the pir, he said he has something with him, a big bad spirit [laughs], that has mixed with his blood. He gave a tabij and recited water; he took it, but nothing helped. He gave tabij, recited water to bathe with, nothing else: the tabij around his neck, arm, and under the carpet of the house [laughs again]. The most helpful thing for my husband has been doctor's medicine.

For many, the psychiatrists were more trustworthy than the mullahs:

The mullahs scare you by saying jadu, jiin, ghosts, etc. in order to make some money out of you. Imams never tell you who has done it. They say naming names would cause fights among the family. I think the real reason they do this is to make money. I do not trust them. I trust the doctors. Initially, the imam did not charge much. Gave us some [blessed] oil. Asked for £150 more. He got worse so we took him [her husband] to the hospital. We did not go back to the mullahs for tabij or anything else.^17^

Only one person, an anglicised young woman, seems to have found anything medical other than pharmaceuticals specifically useful. She tells us in English:

Now if I do lose control, it's only words. [After psychotherapy: before that she used to hit the wall and break objects.] If something is pissing me off, I'll say it is pissing me off, can you just stop it? And because I have had counselling everybody knows that, so when I say stop, they actually stop. When I am under stress I eat a lot. And you know they say with too much stress you get white hair: I do have a lot of white hair. They don't tell me what the tabij is for. All they said is that it would stop anyone doing anything to me and it's trying to cleanse me. They say one of my aunts did something [sorcery] to me, my aunts back home. You know what they are like, ‘Get married’ to their sons, blah, blah. And because I am 25 and I don't get any proposals for me. And their point is ‘why does she always reject and why doesn't any good ones come’? So that means that lady must have done something. They forget the fact that I am small, and teenie weenie and don't talk. And I am pretty much to myself. And marriage proposals … because of that they thought what my aunt did definitely happened.

But virtually all the patients and carers recommended fortitude and faith in God (cf. Littlewood and Dein 2013a). As Rani puts it,

It says in the Qur'an that if you pray with faith, then it will help. I feel that my worries do lessen with prayers. Sometimes the GP gives me some medicine which has no effect whatsoever, and I cry out to God from my heart and pray to be helped and healed; he can choose to take me or heal me. When I say these words, I find comfort and relief, and that then seems to make me much better. I leave all my trust upon God to take care of me. And if I continually just say – that ‘I am in pain, oh the pain’, for instance, then, actually I seem to be in pain a lot more; so prayers seem to bring ease. It's actually what can give peace of mind. If I found a good spiritual healer, I could give it a try. But I have not found any. I say prayers to God; I also send salutations and blessings to the Prophet and then I blow over my chest; I also say *‘Inna-lillahi’*. And I say that God sent me to this world to go through this.

## Conclusion

The dichotomous options between doctor and *tabij* as institutions reflect the broad separation between biomedicine and local healing in Bangladesh (Wilce 2004b). There has been no professionalisation of rural tradition, no middle class moves towards taking up the local as the more ‘holistic’. The relative absence here of references to rural herbalists and diviners (*kobirajs*) suggest that migration has accentuated the ‘modern/local’ divide in the direction of ‘universal’ Islam and ‘universal’ Western science (Gellner 1992; Gardner 1995, 235). Purist Islam in Bengal is generally opposed to hoping for miracles (Gardner 1995, 202–203) but as Gardner (Gardner 1995, 244, 259) notes, in a crisis even Bangladeshis who are supposedly opposed to saintly pirs do visit them. And it can reasonably be assumed that in a migrant (or indeed other) community, mental illness and disability constitute a major crisis.

That migrant communities from developing countries should still use a creolisation of ‘traditional’ and biomedical resources is not unusual. What is perhaps surprising is the high level of trust placed on the rather distant and seldom seen psychiatrist and their biomedical treatments. This is explored further in Littlewood and Dein (2013b). Whilst this may be because the sample was drawn from (day) hospitals, social anthropologists often find that local healers and treatments are morally ambiguous (Callan 2012), and that the healer can even be considered a sorcerer bribed by others, as patients pragmatically try one or the other and then discard them for not being efficacious (e.g. Last 1981) or for even being a witch (Fabrega and Silver 1973). The tabij here, like the mullah, is a potential force for harm as well as good, and pragmatism rules. The doctors though are never considered malevolent: incompetent sometimes, but rarely. Likewise, the suspicions cast on family members, especially in-laws, are common in small-scale communities elsewhere with ‘zero sum economics’^18^ where these relatives are often perceived to be the agents of jealous sorcery: ‘Bengali people’. Callan (2007) comments that ‘in Sylhet there is a tendency to attribute misfortune to other humans’ moral shortcomings – in the context of sorcery, spells and spirits are simply regarded as go-betweens enacting other humans’ malevolence.’ That such suspicions persist in England reflects the recentness of migration and the lack of wider opportunity resulting in the generally closed communities Bangladeshis form in east London (Gardner 1995, 2002, Eade, Peach, and Vamplew 1996).

It became clear, on reading the whole of the transcripts, just how little engagement with the wider society these particular migrants have, whether patients or their families; how their life revolves around jobs in garment factories and small ‘Indian’ restaurants; how even the public space of the local London park is spiritually dangerous, as are members of the opposite sex, whether European or Bengali; and just how restricted their visions and opportunities are besides the mosque, the television and the home. The nuclear households of these unwell London Sylhetis seem particularly isolated compared with the joint households in East Bengal (Gardner 2002). And this is made worse by the crisis of insanity. None of the respondents here critique the politics or racism of the wider British society (cf. Dein, Alexander, and Napier 2008)^19^ or offer non-individualised critiques of society or the economy, but simply offer the personal actions of particular persons (or sometimes ethnic groups). ‘[O]verseas migration leads to new inequalities within the joint household, giving rise to tensions which constitute the perceived increase in sorcery accusations’ says Alyson Callan (2007, 341).^20^ Recent accounts of the ‘modernity’ of sorcery have argued against the earlier anthropological assumption that globalisation and international migration would lead to a speedy reduction in such modes of thought (Geschiere 1997, Comaroff and Comaroff 1999). Whether the fortitude recommended by Islam is a source of strength or merely reluctant fatalism is the subject of another paper (Littlewood and Dein 2013a): if the mullah is often an object of mistrust and suspicion for Bangladeshis, then that is certainly not true of their God.

It is as if those with whom one has fairly regular contact and reciprocal social obligations (family, mullahs) are suspect or unavailing, and the more distant authority figure (doctor, God) is the one on whom one must rely,^21^ the distance serving to demonstrate their representing benign moral principles rather than the compromised actions of individuals each pursuing his or her own interests. As if mental illness with its essentially unknown aetiology can never be made sense of or fully incorporated into meaningful everyday life, can never be anything other but an arbitrary disaster.

## Notes

1. One of the informants – a 35 year old man born in Bangladesh who has been diagnosed as having schizophrenia.

2. The 2001 British census showed that the Bangladeshi population represented 0.5% of the population of England and Wales, and 33.4% (65,553 inhabitants) of the population of Tower Hamlets (east London). Tower Hamlets has the highest unemployment rate in London and the lowest male life expectancy at 72 years (DH 2005). British Bangladeshis have a number of characteristics that mark them out from other South Asian groups (Eade, Peach, and Vamplew 1996): a rapid and continuing growth in its population size; a high proportion of young people; larger family size; a concentration of residents within greater London, especially in Tower Hamlets; a high degree of segregation from other communities; low socio-economic status; high rates of unemployment and dependence on local authority housing. Ninety-two percent of British Bangladeshis are Sunni Muslims and the mosque is central to the community's lives, and people turn to it for informal psychological support, often visiting their religious leaders before seeing a medical practitioner (Dein, Alexander, and Napier 2008).

3. *‘Ufri* [evil atmosphere from jinn or other spirits: cf. Callan 2012] is when you walk underneath a tree maybe and something catches you like say bad air; you can get bad air from the graveyard too. [Some informants said it could not happen in England, others that it did.] But *korni* – sorcery – is when someone does something to you. Azim's wife Jasmin thought it was *korni* because Azim's cousin had wanted him to marry her. But Azim's dad didn't want it, and wanted to go up market and get Azim married to Jasmin; Azim was good-looking at the time, you could sell him because of that. Jasmin's relatives were influential, her uncle was a chairman [of a village council], her family were from a higher class than Azim. Azim's family are lower caste.’ *Jadu* and *korni* are sorcery, sometimes called ‘black magic’ by Bangladeshis in English. Sorcery can happen both in England and (particularly) Bangladesh, or pass from Bangladesh to England (but nobody cited the reverse). Jadu may also be glossed simply as ‘magic’ in that it is not always malevolent (Gardner 1995).

4. Local classifications of illness in small scale communities worldwide generally distinguish insanity from mental handicap which is seen as developmental, for instance ‘madness’ versus ‘doltishness’ in Trinidad (Littlewood 1993). They have more difficulty distinguishing insanity from a naturalistic illness. (Is madness natural or ultrahuman?)

5. Spirits created by God out of fire as humans are created out of mud. They have free will and may be mischievous, evil or benign, may be Muslim or Christian or other, get married and procreate sometimes with humans (to produce similar spirits called *rab*) (Crapanzano 1973, Callan 2005). On the whole, ‘purist Islam’ (like the Wahabi initiatives in Bengal) has a dislike of local possession cults and their particular practice of charismatic exorcism (both common in Bangladesh), but the actual existence of jiin and jiin possession is supported by the Qur'an and thus supposedly unquestionable.

6. An English term used by some informants in Britain for spirits that are generally playful if not really benevolent.

7. *Shaytaan* (Satan): either the personified Devil himself, a malevolent non-Muslim jiini or simply ‘the rebellious part of your personality’ (as one informant put it).

8. *Ghost*: word used by some informants in English to vaguely mean a spirit of some type, but here generally *not* that of a deceased person (compare: *bhut*, a ghostly incubus and *pori*, apparently the female equivalent).

9. Religious school.

10. *Tabij*: amulet containing Qur'anic verses or other mantra usually prepared by a clergyman (or indeed by anyone who claims to have the knowledge).

11. *Pora pani*: ‘recited water’, water over which Qur'anic verses are quietly recited. It may then be drunk or sprayed over the person or, like recited oil, be used as an embrocation.

12. *Pak*: in a state of purified body and spirit achieved by prayer and ritual cleansing with water.

13. *pir*: ‘living saint’, mystic, a revered Sufi: religious figure in South Asia, often charismatic and usually with the power to offer spiritual counselling and healing greater than an imam: of a ‘diverse body of holy men’ (Callan 2008, 398), a heterogeneous group including both the purists and the more ecstatic. Although shading into each other at the popular level, the *imam* is originally the ‘caller of prayers’ in the mosque (although often with higher spiritual and social status especially among Shia); the *mullah* is one with at least three years education in a madrasha and who can recite the Qur'an by heart (Callan 2008, 398); the *measaab* is one with some religious knowledge, a dedicated student at a madrasha; a *kobiraj* is a local herbalist or bone setter usually of low status; and a *murshid* is another saintly figure who may act as a guru.

14. It seems uncertain whether, psychiatrically speaking, this was a hallucination, a delusional misinterpretation or a veridical experience: the point here is the interpretation.

16. Although up to the early twentieth century Sylhet was administered together with Assam not East Bengal, Sylhetis may refer to themselves as ‘Bengalis’: the two languages are of course closely related.

17. Clearly, the setting of the interviews (community and mental health centres) and the perceived identity of the interviewers (community workers associated with the health services) might have angled responses in a particular direction favourable to biomedicine; and the Sylheti identity of the two interviewers might have encouraged more critical comments about the local Bangladeshi clergy and residents (‘we Bengalis…’). Or might not. As Wilce (1995) notes, the content and style of any communication depends on the recognised audience and their responses; he describes a wide variety of styles of complaint and responses to them in Bangladesh (Wilce 2004b).

18. Where, as in warfare, one person's gain or success is inevitably another's loss: taking the whole community together, there is a zero sum. Individual success leads to suspicions of unfair means such as sorcery. Even migration can be associated with real economic losses as well as gains for the family back in Bangladesh (Gardner 1995). The rather vague suspicions here of malevolent sorcery from some of our informants obviously miss the full context in which they have developed: particular kinship and economic relations between people, the relative prestige of families, the individual personalities involved, the particular point in time when the suspicion is mooted. Sorcery accusations only make sense with a greater knowledge of the context and contingencies in which they develop; the accounts here omit the ‘functionality’ of sorcery accusations by isolating them off from social life.

19. Although four informants in their turn made critical remarks about specifically African or African Caribbean doctors and social workers (‘black’).

20. Bangladesh itself has of course been changing since the migrants left but the common perception is that the country remains extremely poor, dependent on other countries and on remittances home (Gardner 1995, Callan 2012).

21. ‘Purist’ Saudi Islam (scriptural, clerical, ritualistic) which has become increasingly influential in Bangladesh opposes itself to the ‘magical’ Islam of Bangladesh, which is regarded as both Sufi (mystical) and influenced by Hinduism. It is enthusiastic about technological modernity including biomedicine (Gardner 1995, 235). Hence, older Bangladeshi practices (pirs, tabij, kobiraj, miracle seeking) can be seen as ‘squeezed out’ by Western medicine and revitalist Islam, both here acting in the same ‘modern’ direction. As far as the authors are aware, there are no kobiraj healers in London, although bits and pieces of their practice are still continued by lay people and clergy.
